# Biological and behavioral markers of pain following nerve injury in humans

**DOI:** 10.1016/j.ynpai.2019.100038

**Published:** 2019-12-04

**Authors:** S.A. Holmes, N. Barakat, M. Bhasin, N.I. Lopez, A. Lebel, D. Zurakowski, B. Thomas, S. Bhasin, K.E. Silva, R. Borra, R. Burstein, L.E. Simons, D. Borsook

**Affiliations:** a Department of Anesthesiology, Critical Care & Pain Medicine, Boston Children’s Hospital, Boston, MA 02215, United States; b Department of Anesthesia, Harvard Medical School, Boston, MA 02115, United States; c Department of Anesthesiology, Perioperative & Pain Medicine, Stanford University School of Medicine, Stanford, CA 94305, United States; d Department of Radiology, University Medical Center Groningen, University of Groningen, Hanzeplein 1, 9713 GZ Groningen, Netherlands; e Department of Nuclear Medicine and Molecular Imaging, University Medical Center Groningen, University of Groningen, Hanzeplein 1, 9713 GZ Groningen, Netherlands; f Department of Anesthesia, Critical Care and Pain Medicine, Beth Israel Deaconess Medical Center, United States; g Bioinformatic and Systems Biology Center, Beth Israel Deaconess Medical Center, United States; h Department of Medicine, Harvard Medical School, United States

## Abstract

The evolution of peripheral and central changes following a peripheral nerve injury imply the onset of afferent signals that affect the brain. Changes to inflammatory processes may contribute to peripheral and central alterations such as altered psychological state and are not well characterized in humans. We focused on four elements that change peripheral and central nervous systems following ankle injury in 24 adolescent patients and 12 age-sex matched controls. Findings include (a) Changes in tibial, fibular, and sciatic nerve divisions consistent with neurodegeneration; (b) Changes within the primary motor and somatosensory areas as well as higher order brain regions implicated in pain processing; (c) Increased expression of fear of pain and pain reporting; and (d) Significant changes in cytokine profiles relating to neuroinflammatory signaling pathways. Findings address how changes resulting from peripheral nerve injury may develop into chronic neuropathic pain through changes in the peripheral and central nervous system.

## Highlights

• Integrating genetic analyses, clinical testing and MRI (PNS/CNS).
• Early stages of pain development in a group with confirmed neuropathic pain.
• MRI changes in peripheral nerve fibers resulting from ankle sprain.

## 1 Introduction

The dynamic interplay between the peripheral and central nervous system contributes to the phenotypic expression of the evolution and devolution of chronic pain. For some painful conditions, there is an obvious inciting event that produces neuropathic pain, which may be peripheral (e.g., trauma) or central (e.g., spinal cord injury) in its origin. In those cases of peripheral nerve damage, ongoing processes such as ectopic firing of damaged nerves (Serra, 2012, Sun et al., 2005) or peripheral sensitization from an ongoing inflammatory response (Ellis and Bennett, 2013, Ji et al., 2018, Schafers et al., n.d..) may be the harbinger of changes in the central nervous system (Apkarian and Reckziegel, 2018, Woolf, 2011), resulting in pain chronification (Hashmi et al., 2013) and persistence as a consequence of changes in sensory, emotional, and other brain networks (Borsook et al., 2018, Hemington et al., 2016).

An example of chronic pain that evolves following tissue or nerve injury is ankle sprain (Ogilvie-Harris et al., 1997). Here an eversion or inversion of the foot may produce a stretch injury, usually involving nerves in the foot contralateral to the inversion or eversion process. There are an estimated incidence rate of 2.15 per 1000 person-years of ankle sprains in the United States each year, with 75–85% of these being ankle sprains and the majority in the 10–19 yr age range and no sex difference (Waterman et al., 2010). Following ankle sprain, injury to the tibial and more commonly the fibular nerve, is common and can occur in up to 80% of cases (Hunt, 2003). While most recover in the months following the injury, up to 40% can have chronic symptoms (Safran et al., 1999). Stretch injuries associated with ankle sprains can result in neuropathic pain due to nerve stretching or microscopic tears in ankle ligaments (Hubbard et al., 2016, Menorca et al., 2013). An ankle sprain can set off a cascade of events including pain, inflammatory response, and inactivity (Ellis and Bennett, 2013, Littlejohn, 2015). Typical neuropathic features include burning pain, electric-sensation pain, tingling, and numbness (Baron et al., 2017, Colloca et al., 2017). At the ankle, branches of the Tibial (medial and lateral planter nerve of the foot) and Fibular (peroneal) nerves are involved; these two nerves contribute to the formation of the sciatic nerve (Giuffre and Jeanmonod, 2018). The incidence of nerve damage varies across studies and depends on the grade of sprain. The duration of neuropathic pain following these injuries is not well documented in adults or children (van Ochten et al., 2014) but may serve as an ideal model to evaluate neuropathic evolution and devolution over time.

**Fig. 1:**
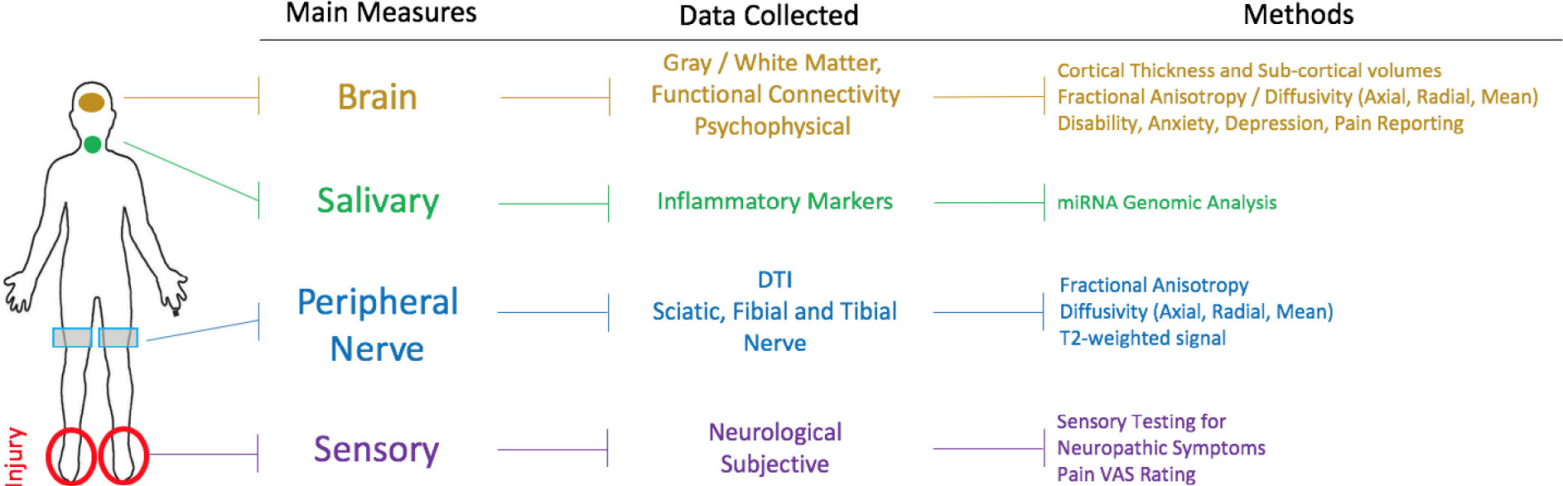
Summary of study. Diagram showing the different sites of evaluation used in the current investigation and the location of injury. For each measure, a list of the extracted parameters is outlined as well as the methods used.

How peripheral nerve injury, initially producing acute pain-related dysfunction, may progressively alter brain function that induces the pain phenotype to include sensory, affective, and cognitive changes is currently not well understood. Most clinical studies of chronic pain focus on the brain or peripheral effects but not both. Here we use the ankle injury model to evaluate peripheral nerve injury induced pain to evaluate the evolution of pain phenotype in three processes: peripheral nerve measures, inflammatory markers following injury, and central nervous system markers of pain. *Our overall hypothesis is that nerve damage from ankle injury with features of neuropathic pain is associated with elevated inflammatory responses and brain changes concordant with the resultant disability and elevated level of pain symptoms.* Our approach is consistent with recent suggestion of utilizing composite measures to evaluate markers of disease (Tracey et al., 2019) and the results indicate that multiple measures (i.e., psychological, nerve, brain and inflammatory markers) in the clinical condition may provide a more accurate evaluation and mechanistic understanding of drivers of pain evolution or devolution following nerve injury. A visual summary of measures can be found in Fig. 1.

## 2 Methods

### 2.1 Participants

This study recruited participants from the Boston and the Greater Boston area. A total of 24 patients and 12 healthy controls were recruited. Each clinical subject was diagnosed with peripheral neuropathy from a clinical assessment performed by a project-affiliated physician (DB or AL). Inclusion criteria was otherwise healthy individuals ages 10–24 who present with a unilateral lower extremity sprain injury and evidence of neuropathic pain as confirmed through medical evaluation by a project-affiliated physician. Exclusion criteria included: claustrophobia, significant medical problems (e.g., uncontrolled asthma, seizures, cardiac disorder), drug use (e.g., opioids, marijuana), psychiatric problems (e.g., active suicidality), and other neurological disorders, pregnancy and any device or medical concern that preclude being scanned using an MRI (e.g., magnetic implant, exceeding weight limit of scanner). Healthy controls were recruited through flyers on bulletin boards, online advertisements, and word of mouth and underwent the same diagnostic and screening procedures as patients. Healthy controls could not have any history of ankle sprain within the past three years prior to enrollment. All participants were compensated for their time. Written informed consent was obtained for all procedures, which were approved by the Boston Children’s Hospital. A list of what data was available for which modality is provided in Supplementary Table 1.

### 2.2 General statistical considerations

For our sample size calculation, we used the most variable source of data collection – the DTI measures of the peripheral nerve. Effect size for peripheral nerve damage measured against the contralateral side is 1.67. To detect changes that are at least 30% of the observed/published ones with an effect size of 0.5, alpha of 0.05 and 80% power 24 patients are required.

### 2.3 Neurological examination

A medical evaluation involving review of medical history and pain symptoms, and a detailed neurological examination were performed by trained study clinicians to confirm the presence of neuropathic pain. The exam evaluated the area of injury for indications of peripheral nerve injury such as the presence of allodynia and atypical physiology (e.g., abnormal sweating). The exam also confirmed patient reported date of injury and pain intensity using a visual analogue scale.

### 2.4 Questionnaires

All subjects completed a battery of questionnaires relating to pain. Questionnaires included those relating to pubertal development, pain-related functional limitations, pain-related distress, and general distress. Initial sample characterization was performed using the Pubertal development scale (PDS) (Peterson et al., 1988). Pain-related disability was evaluated using the Functional Disability Inventory (FDI) (Walker and Greene, 1991). Pain related effects were evaluated using the Pediatric pain screening tool (PPST) (Simons et al., 2015) Fear of pain questionnaire (FOPQ) (Simons et al., 2011) and Pain catastrophizing score (PCS) (Sullivan, 1995). Anxiety and depression levels were evaluated using the Multidimensional Anxiety Scale for Children-2nd Edition (MASC-2) (March et al., 2013) and Children’s Depression Inventory-2nd Edition (CDI-2) (Kovacs, 2010). Questionnaires were recorded without pain provocation. Data was missing for one ankle sprain participant on the PPST, PCS, CDI, FDI, and FOPQ.

### 2.5 Targeted transcriptome profiling on saliva samples

Target Transcriptional profiling of 23 adolescents with ankle injury and 12 matched controls was conducted using the digital multiplexed nCounter® GX Neuroinflammation kit containing 770 Neuroinflammation related genes. Normalizations and quality controls were performed using R packages (Angin et al., 2014, Perry et al., 2016). Unsupervised analysis was performed on normalized data using Principal Component Analysis and hierarchical clustering analysis. Differentially expressed genes were identified using Limma: linear model microarray analysis software package where genes were ranked by t-statistic using a pooled variance (Ritchie et al., 2015). Differentially expressed genes (DEG) were identified on the basis of false discovery rate (FDR) adjusted p-value and Fold change. The FDR was controlled using the Benjamini and Hochberg algorithm. The pathways, functions and interactive networks analysis were performed on DEG using Ingenuity Pathways Analysis (IPA 9.0) (http://www.ingenuity.com/) package.

#### 2.5.1 Pathway and interactive network analysis

Ingenuity Pathway Analysis (IPA 9.0, Qiagen) was used to identify the pathways and interaction networks that are significantly affected by significantly differentially expressed genes. The knowledge base of this software consists of functions, pathways and network models derived by systematically exploring the peer reviewed scientific literature. A detailed description of IPA analysis is available at the Ingenuity Systems’ web site (http://www.ingenuity.com). It calculates a p-value for each pathway according to the fit of users’ data to the IPA database using one-tailed Fisher exact test. The pathways with adjusted p-values <0.05 were considered significantly affected. For each network, IPA calculates a score derived from the p-value of one-tailed Fisher exact test [score = −log(p-value)] and indicates the likelihood of focus genes appearing together in the network due to random chance. A score of 2 or higher has at least a 99% probability of not being generated by random chance alone. The ability to rank the networks based on their relevance to the queried data sets allows for prioritization of networks with the strongest association with injury group.

#### 2.5.2 Regulatory module analysis

The regulatory module analysis was used to identify the cascade of upstream transcriptional regulators that can explain the observed gene expression changes to help identify key regulators (master regulators) and understanding underlying biological mechanism (Ritchie et al., 2015). The analysis will help in identifying first which transcription regulators are significantly affected in injury group as compared to control as well as determining whether they are activated or inhibited. The activation or inhibition of transcriptional regulators was determined by determining the overlap among user’s data with activation or inhibition signatures of regulators. The significance of overlap was determined using one-tailed fisher Exact test. For further information relating to methods please see (Perry et al., 2016).

### 2.6 Magnetic resonance imaging

All subjects were scanned using a 3T Siemens Magnetom Trio scanner (Siemens Healthcare Inc., East Walpole, MA, USA) located at Boston Children’s Hospital in Waltham, Massachusetts. Anatomical positions are reported in Talairach units.

#### 2.6.1 Peripheral nerve imaging

Peripheral nerve imaging was performed using a 15-Channel knee coil. In order to optimize image quality, the scanner leg was positioned as close as possible to the isocenter while avoiding angulation of the leg with respect to the Z-direction of B_0_. DTI images were obtained at a standardized location over the knee (10 cm), using the upper border of the patella as an anatomical reference. Data were missing from one leg for two healthy controls and two patients. The imaging protocol included T2-weighted imaging, Diffusion Tensor Imaging (DTI) (FOV = 164 × 48mm^2^, Acquisition time (TA)/Repetition time (TR)/Echo time (TE) = 6.14 min/4400 ms/103 ms, 20 diffusion directions, 4 averages, b-value = 0 s/mm^2^, 750 s/mm^2^, voxel size: 0.8 × 0.8 × 5 mm^3^). Imaging analysis was performed using Olea SphereTM V2.3 (Olea Medical, Paris, France) that included motion-correction as part of a standard pipeline. The sciatic nerve was localized using T2-weighed images in both legs. In this way, the unaffected limb served as a control for the same patient’s affected leg, as well as a control to then compare with healthy controls. Regions of interest were drawn at the sciatic, tibial and fibular levels on DTI maps and were verified by a second reviewer. Fractional anisotropy (FA), mean diffusivity (MD), radial diffusivity (RD), axial diffusivity (AD) and T2-weighted signal were extracted for analysis.

#### 2.6.2 Structural brain imaging

Brain imaging was performed using a 32-Channel head coil. T1-weighted anatomical images were acquired using a multi-echo acquisition (TA = 7.37 min; TR = 2520 ms; TE: 1.74/3.6/5.46/7.32 ms, 651 Hz/Px, FOV = 240 × 240 mm^2^, slice thickness of 1 mm, 176 slices, voxel size: 1 × 1 × 1 mm^3^, TA = 5:50. Diffusion tensor imaging was acquired using the following parameters: TA/TR/TE = 7.53 min/5200 ms/109 ms, Slice Thickness = 2 mm, 70 slices, bandwidth 1666 Hz/Px, FOV = 240 × 240 mm, voxel size: 2 × 2 × 2 mm^3^, b-value = 2000 s/mm^2^, 81 directions. Freesurfer (http://surfer.nmr.mgh.harvard.edu) was used for pre-processing of T1-weighted images that included motion correction, intensity normalization, skull stripping, white and grey matter segmentation and cortical parcellation. Grey matter thickness was evaluated using the qdec program in Freesurfer with spatial smoothing of 10 mm FWHM and an FDR threshold of p = 0.05 (Fischl and Dale, 2000). Pre-processing DTI data included eddy-current correction, head motion correction, intra- and inter-subject registration, and tensor fitting. DTI parameters were extracted from eighteen major white matter tracts: Forceps Major, Forceps Minor, and bilateral Anterior Thalamic Radiation (ATR), Cingulum – Angular Bundle (CAR), Cingulum – Cingulate Gyrus (CCG), Corticospinal Tract (CST), Inferior longitudinal Fasciculus (IFL), Superior Longitudinal Fasciculus – Parietal (SLF-P) and Temporal (SLF-T) division, and Uncinate Fasciculus (UNF). Findings from the DTI analyses were evaluated between groups in the pre-defined tracts of interest at the level of p = 0.05 using an independent sample *t*-test.

#### 2.6.3 Functional brain imaging

Resting state functional neuroimaging was acquired from 23 ankle injury patients and 10 healthy controls. Imaging parameters included: TR/TE: 1110/30 ms, slice thickness 3 mm, FOV: 228 mm × 228 mm^2^, voxel size: 3 × 3 × 3 mm^3^, number of slices = 51, TA = 7:59. Resting state fMRI was processed using the functional connectivity toolbox (CONN toolbox) (Whitfield-Gabrieli and Nieto-Castanon, 2012) implemented in MATLAB (9.4: The Mathworks, Natick, MA, USA). Preprocessing of images included realignment, field map correction, slice timing correction, image co-registration, image normalization, outlier and spatial smoothing (8 mm). Extracted data included global efficiency, local efficiency, betweenness centrality, cost, average path length, clustering coefficient, and degree. A false discovery rate of 0.05 was used for seed-to-voxel connectivity as well as graph theory analysis.

#### 2.6.4 Statistical analysis

Statistical analyses were performed using R (R Core Team, 2019). Whole-brain analyses were performed with an FDR correlation factor of p = 0.05 and group comparisons were performed using independent sample t-tests. Peripheral nerve data were submitted to a model evaluating the interaction between group (HC, Ankle injury) and Nerve (Sciatic, Tibial, Fibular) while controlling for which leg was evaluated (left, right) and the injured leg (left, right). Interactions were decomposed by evaluating group effects in each nerve segment, controlling for evaluated leg and injured leg. Data are reported as mean +/− standard deviation unless otherwise noted. Finally, in an attempt to integrate study findings, we perform a cross-correlation analysis between all study variables to discern trends and patterns between the healthy control and ankle sprain cohorts and review results qualitatively.

## 3 Results

### 3.1 Demographics and psychological findings

No group differences in age (Healthy Control: M = 17.33 SD = 4.08; Ankle Injury: 16.93 SD = 3.74; t = 0.29, p = 0.771) or gender (Healthy controls: 7 Female; Ankle Injury: 13 Female); χ^2^ = 0.06, p = 0.813) were observed. Overall, seven individuals from the ankle injury group had right-sided injuries and 17 had left-sided. Average time since injury was 33 days (SD 18.98). No group difference was found on the CDI (Healthy Control: M = 43.25 SD 4.59; Ankle Injury: M = 46.78 SD 5.62) p = 0.053, the MASC (M = 40.75 SD 1.42; M = 42.83 SD 4.53) p = 0.237, or on the PDS (Healthy Control: M = 17.83 SD 3.70; Ankle Injury: M = 15.79 SD 4.00), p = 0.15). Patients reported a maximum level of pain intensity of 8.04 (Range = 3–10) an average level of pain of 2.54 (Range = 0–7) and were on average 33 days (SD = 18.98; Range = 10–89) from their injury.

### 3.2 Peripheral measures

#### 3.2.1 Peripheral nerve profile

**Fig. 2:**
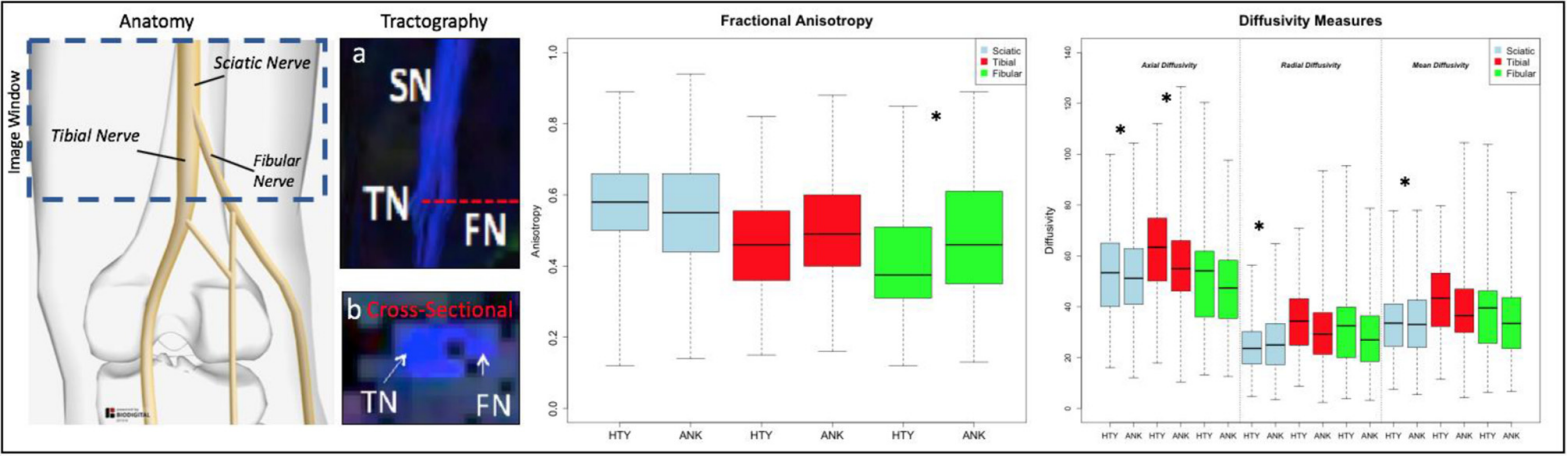
Peripheral nerve measures. (Left) A schematic is outlined showing the recording window and its position within the leg as well as representations of the sciatic, tibial and fibular nerves using schematic and tractography representations. Recordings were taken from both the left and right legs. (Middle) Findings from the diffusion weighted imaging analysis using fractional anisotropy as well as other diffusion measures (Right). * Indicates a significant difference (p < 0.05) in a regression model controlling for the recorded leg (left or right) and the location of injury (left or right ankle). HTY = Healthy; ANK = Ankle Injury.

DTI parameters of FA, MD, AD, and RD, as well as T2 signal were extracted from the Sciatic, Tibial and Fibular nerves. Results can be found in Table 1, and Fig. 2. Significant interactions between Group and Nerve were found for FA, RD, MD and AD (p < 0.05). Both main effects of Group and Nerve were significant for T2-weighted signal with a non-significant interaction term (p = 0.617). Post-hoc analyses showed that a main effect of group was observed in the Fibular nerve for FA (HC < Ankle), Tibial nerve for AD (HC < Ankle), and in the Sciatic nerve for RD (HC > Ankle), MD (HC < Ankle), and AD (HC < Ankle).

**Table 1:** Peripheral nerve model results. Findings from the omnibus full model tests evaluating for Group (Healthy Control; Ankle Injury) by Nerve (Sciatic; Tibial; Fibular) interactions. Significant interactions (bold) were evaluated using an ANCOVA model if they were below the p = 0.05 threshold. All models were controlled for which leg was evaluated and which leg was injured.

Full Model					Post-hoc Analysis					
					Sciatic		Tibial		Fibular	
		F-value	p-value		F-Value	p-Value	F-Value	p-Value	F-Value	p-Value
Fractional Anisotropy	*Group*	**4.26**	**0.039**							
	*Nerve*	**18.39**	**<0.001**							
	*Group × Nerve*	**11.28**	**<0.001**	*Group*	0.664	0.415	0.373	0.542	**7.157**	**0.008**

Radial Diffusivity	*Group*	**20.05**	**<0.001**							
	*Nerve*	**45.13**	**<0.001**							
	*Group × Nerve*	**8.79**	**<0.001**	*Group*	**7.188**	**0.007**	2.42	0.121	3.216	0.074

Mean Diffusivity	*Group*	**24.02**	**<0.001**							
	*Nerve*	**33.76**	**<0.001**							
	*Group × Nerve*	**6.38**	**0.002**	*Group*	**13.951**	**<0.001**	3.176	0.076	1.601	0.207

Axial Diffusivity	*Group*	**29.97**	**0.001**							
	*Nerve*	**25.78**	**0.001**							
	*Group × Nerve*	**3.09**	**0.046**	*Group*	**27.505**	**<0.001**	**4.222**	**0.041**	0.288	0.592

T2 Signal	*Group*	**14.36**	**<0.001**							
	*Nerve*	**134.70**	**<0.001**							
	*Group × Nerve*	0.48	0.617

### 3.3 Neuroinflammation genes profile

**Fig. 3:**
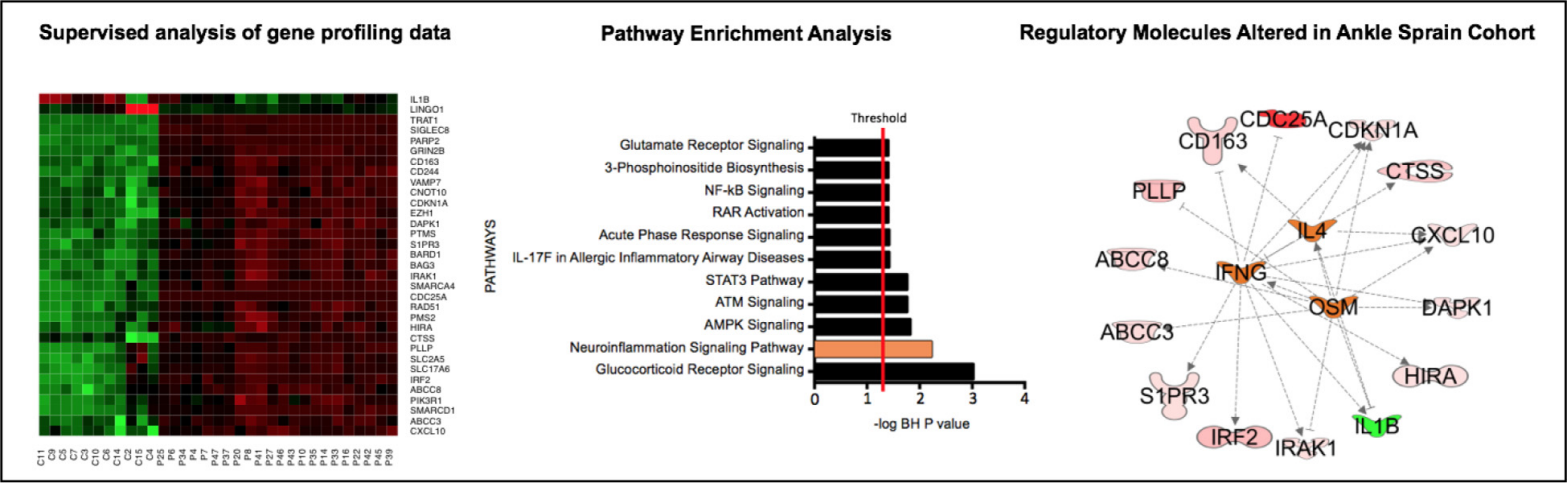
Genetic profiling analysis. (A) Heat map of 33 differentially expressed genes from saliva of injury patients vs. control subjects. Supervised analysis of gene profiling data from ankle injury patients and control subjects. The genes with multiple test corrected p value <0.05 and absolute change >2 folds were considered significantly and differentially expressed. In each heat map, rows depict differentially expressed genes and columns depict individual subjects. The relative expression level of genes is shown using a pseudocolor scale from −3 to +3 (green represents down regulation and red represents up regulation). (B) Pathways enrichment analysis of genes that are differentially expressed in saliva from ankle injury patients compared to controls. This analysis depicts the significant effect on multiple stress and inflammation pathways including DNA Damage, Neuroinflammation, AMPK and ATM signaling. Pathways with multiple test corrected p value <0.05 were considered significant. (C) Top regulatory molecules (orange) significantly altered in injury group as compared to controls. Lines ending in an arrow represent amplification and those ending in a flat line represent an inhibitory connection. (For interpretation of the references to color in this figure legend, the reader is referred to the web version of this article.)

Unsupervised analysis was performed on neuroinflammation genes expression profile data after normalization from high quality saliva samples data after normalization using principal component analysis. The analysis revealed two distinctive clusters of saliva data (Ankle injury study vs Controls) based on expression of these inflammation related genes (Fig. 3), consistent with the two clinical groups.

Supervised analysis based on absolute fold change ≥2, and multiple test corrected P value <0.05, revealed 33 genes differentially expressed between Ankle injury and control groups (Fig. 3). Heatmap depicted a consistent segregation between Ankle injury and control groups based on expression of these genes. The analysis identified significant upregulation of multiple Mitochondrial ATPase (i.e. DAPK1, ABCC8, VAMP7), Cytokine, interleukin, Defense and immune response genes (i.e. IRAK1, DAPK1, IRF2, GRIN2B, CD244, TRAT1, CXCL10, S1PR3, CTSS, CD163, VAMP7) in the Ankle injury group. Further pathway analyses on these differentially expressed genes depicted significant enrichment (Adjusted P value <0.05) in multiple stress and inflammation pathways including DNA Damage, Neuroinflammation, AMPK and ATM signaling (Fig. 3**-**a). Interestingly Neuroinflammation pathways depicted significant activation in Ankle injury group (Z-score = 1.3, p-value <0.01). Further upstream key regulator analysis on differentially expressed genes indicated significant activation of IL4 (Z-score = 1.5), IFNg (Z-score = 1.6) and OSM (Z-score = 1.5) regulated genes (Fig. 3**-**b). Additionally, Scale-free Interactive network analysis identified multiple genes that formed regulatory or highly connected nodes that serve as focus hubs in the networks and might be crucial for pathogenesis (Fig. 3**-**c). The focus hubs are formed by inflammation regulated genes (e.g., TNF-α, NFκB, TGF-β, CXCL10), kinases (e.g. AKT, PI-3 Kinase) and cell cycle proliferation related genes (CDKN1A, ERK).

### 3.4 Central measures

#### 3.4.1 Brain profile

##### 3.4.1.1 Cortical thickness and sub-cortical brain volumes

**Fig. 4:**
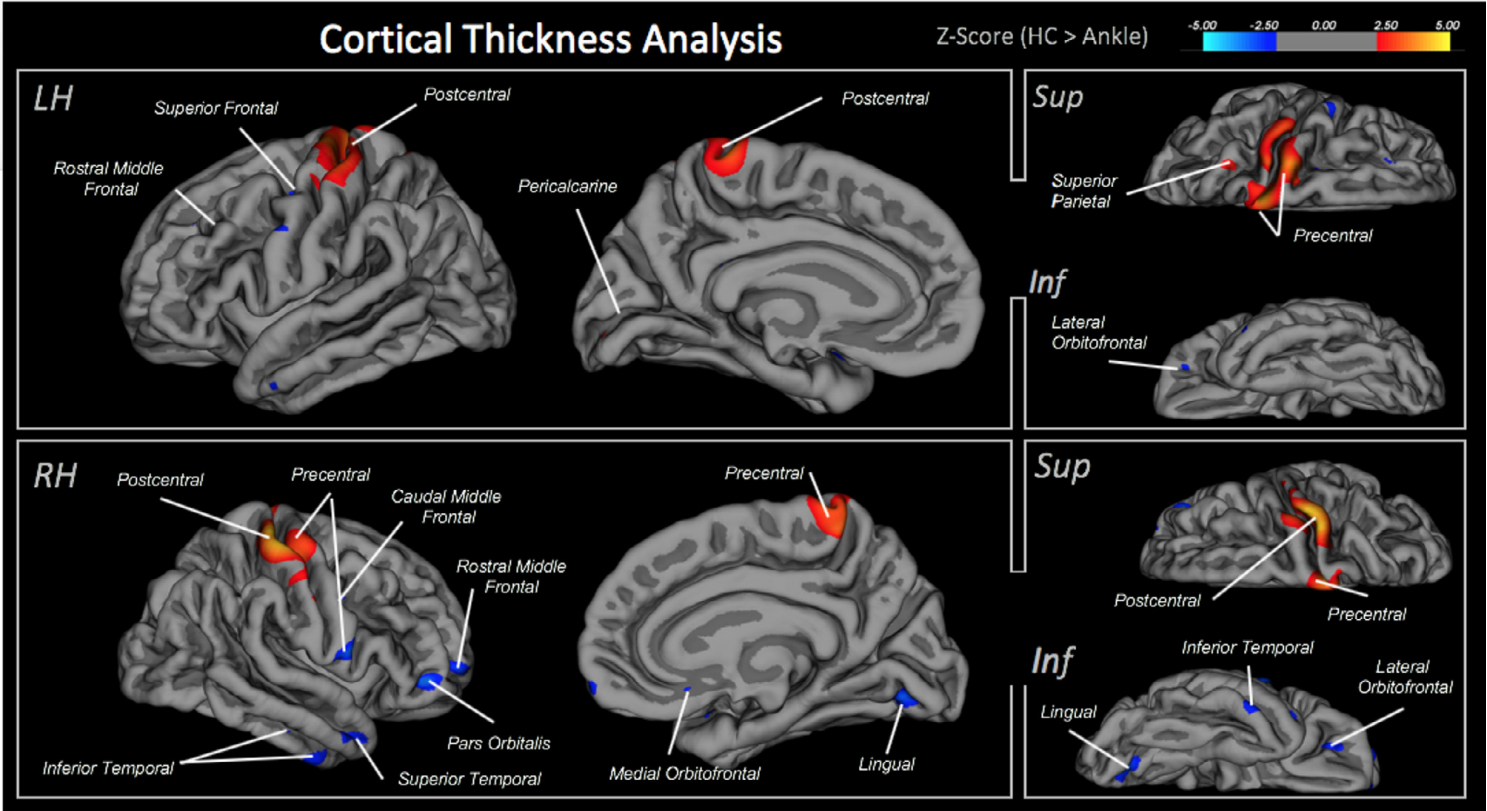
Cortical thickness measures. Comparing cortical thickness between the healthy control and ankle injury cohorts. Each highlighted brain region depicts a significant difference between subject groups at the FDR-corrected level of 0.05. Red = greater thickness in healthy controls; Blue = greater thickness in ankle injury cohort. All significant brain regions are ported in the table below along with the size of the cluster and center of gravity reported in Talairach coordinates. (For interpretation of the references to color in this figure legend, the reader is referred to the web version of this article.)

Findings from the cortical thickness analysis showed significant group differences (see Fig. 4). In the left hemisphere, greater thickness was found in healthy controls relative to the ankle injury cohort in the precentral and postcentral gyrus and superior parietal cortex. Greater cortical thickness was found in the control group in the isthmus cingulate, superior temporal, lateral orbitofrontal, rostral middle frontal, entorhinal, precentral, and pars opercularis. In the right hemisphere, greater thickness was found in Healthy controls in the post- and precentral gyrus; whereas greater thickness was found in the Ankle injury cohorts in regions including the rostral and caudal middle frontal gyrus, insula, lingual, and superior and inferior division of the temporal cortex (Fig. 4). No differences in sub-cortical volumes were observed.

##### 3.4.1.2 Brain diffusion tensor imaging – Tractography

**Fig. 5:**
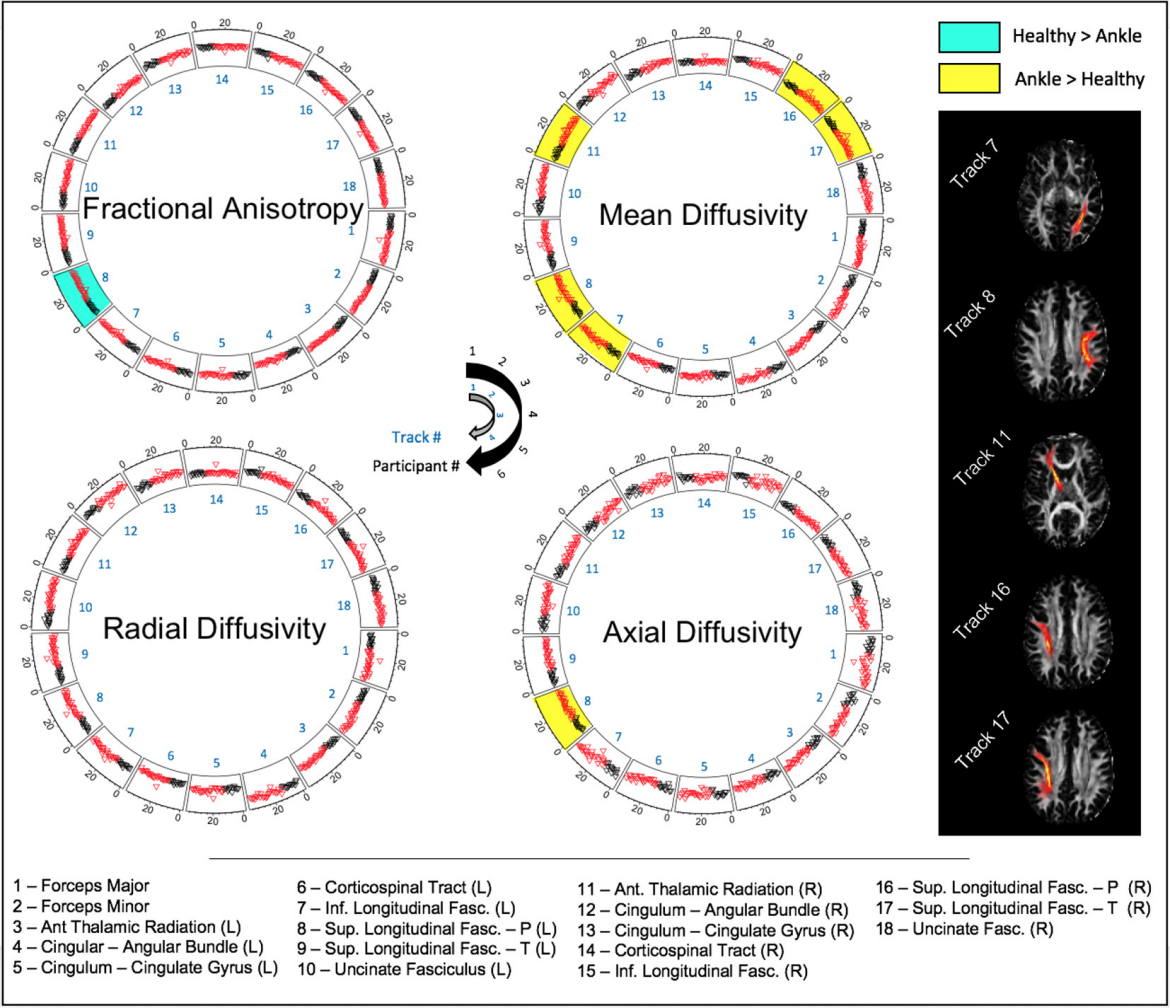
Circular diagrams presenting tractography of white matter pathways. Significant differences between healthy control and ankle injury cohorts are highlighted within each ring. Rings represent output from all 18 white matter pathways (inside of ring) with individual points reflecting subject values (outside of ring). White matter tracts (right side) from an exemplar subject show the five tracts with significant group differences. R = right hemisphere; L = Left hemisphere.

Tractography was performed on eighteen major white matter tracts in the right and left hemispheres of the brain (see Fig. 5). Five tracts showed significant findings. Mean diffusivity was found to be higher in the ankle injury cohort in the LH-IFL, t(34) = 2.218, p = 0.033, LH-SLF-P, t(34) = 2.282, p = 0.029, and RH-ATR, t(34) = 2.065, p = 0.047, and RH-SLF-P, t(34) = 2.466, p = 0.019, and RH-SLF-T, t(34) = 2.134, p = 0.04. Fractional anisotropy was lower in the ankle injury cohort in the LH-SLF-P, t(34) = 2.066, p = 0.047. Radial and axial diffusivity were found to be higher in the ankle injury cohort in the LH-SLF-P, t(34) = 2.245, p = 0.031, and RH-SLF-P, t(34) = 2.153, p = 0.038, respectively. All other tracts were not significant (p > 0.05).

#### 3.4.2 Resting state functional connectivity

Graph theory was used to evaluate whole brain function. Between the two groups, there were no differences in terms of global efficiency, betweenness centrality, average path length, and degree. Only the posterior aspect of the inferior temporal gyrus in the left hemisphere was found to show a significance difference with greater local efficiency, beta = −0.06; t(31) = 4.50, p-FDR = 0.012 and clustering coefficient, beta = −0.10; t(30) = −4.67, p-FDR = 0.007. Resting state seed-to-voxel connectivity was performed in three a priori regions of interest from regions shown to be implicated in pain and fear of pain (see Simons et al., 2016) within the amygdala, nucleus accumbans (NAc), and periaqueductal grey (PAG). Decreased functional connectivity was found between bilateral PAG and the frontal pole as well as the right NAc and a cluster within the frontal oribital cortex/Insula. A cluster showing increased connectivity approached significance between the left amygdala and the superior parietal lobule, post central gyrus and supramarginal gyrus.

### 3.5 Functional limitations, pain-related distress, and general distress

The ankle injury cohort had elevated levels on the functional disability inventory (Healthy control: M = 0.08 SD 0.29; Ankle injury: M = 9.04; SD7.60), t(33) = 4.034, p < 0.001, pediatric pain screening tool (Healthy control: M = 0.08 SD 0.29; Ankle injury: M = 1.83; SD1.88), t(33) = 3.179, p < 0.001, fear of pain questionnaire (Healthy control: M = 9.50 SD 11.60; Ankle injury: M = 22.13; SD15.29), t(33) = 2.503, p = 0.02, and pain catastrophizing score (Healthy control: M = 4.75 SD 5.55; Ankle injury: μ = 12.90; SD8.80), t(33) = 2.903, p = 0.01.

### 3.6 Cross-modal integration

**Fig. 6:**
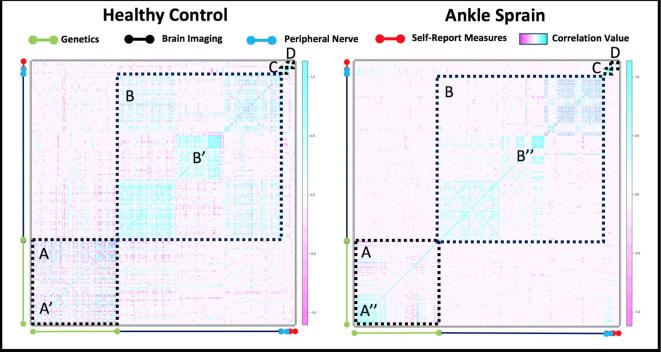
Integrated Cross Modal Analysis. Coherence/correlation maps indicating a relationship between brain or inflammatory marker or self-reported measures (healthy vs. patients). The analysis shows differences in patterns within the heatmap for genes evaluated (A), structural brain regions (B), peripheral nerve measures (C) and psychological/self-reported measures (D). A stronger relationship is indicated by the increase in (blue-purple) vs. a weaker relationship (no-color). (For interpretation of the references to color in this figure legend, the reader is referred to the web version of this article.)

A Pearson-based cross-correlation analysis was performed integrating study data from each domain. As shown in Fig. 6, the map highlights within modal findings including the localized increase in inflammatory molecules (A vs A′) and a shift in gray matter thickness in persons with ankle injury (B vs B′). A comparison between cohorts suggests that there may be an interaction between brain and peripheral nerve imaging with genetic markers as there is a stronger correlation value in the ankle sprain relative to healthy control group. In the ankle injury cohort, we extracted variables with a very high correlation value to understand cross-modal relationships. Using a Pearson correlation value of 0.95 as a cut-off, features reflecting genetics and brain (cortical, and white matter variables) were observed with no findings from the peripheral nerve or self-report questionnaires (see Table 2).

**Table 2:** Correlation extraction. After reducing features based on their Pearson correlation value, this table lists the features that showed a high correlation (r > 0.95). MD – Mean Diffusivity; RD – Radial Diffusivity; FA – Fractional Anisotropy; LH – Left Hemisphere; RH – Right Hemisphere; Sup – Superior; Par – Parietal; Temp – Temporal.

Immunological	Cortical Volumes/Thickness	White Matter	Peripheral Nerve	Psychological
BAG3	Brain Segmentation Volume	MD – Sup.Longitudinal Fasciculus (Par) – RH	–	–
BARD1	Cerebral White Matter Volume	RD_ Sup.Longitudinal Fasciculus (Par) – RH		
CDC25A	Cortex Volume	RD_ Sup.Longitudinal Fasciculus (Temp) – RH		
CDKN1A	Left Hemisphere Mean Thickness	RD_ Sup.Longitudinal Fasciculus (Par) – LH		
Grin2b	Left Hemisphere Cortex Volume	RD_ Sup.Longitudinal Fasciculus (Temp) – LH		
IRF2	Right Hemisphere Cerebral White Matter	FA_Forceps Major		
PARP2	Right Caudate	FA_Sup.Longitudinal Fasciculus (Temp) – LH		
PLLP	Supratentorial			
SIGLEC8	Total Gray Matter Volume			
SMARCA4				
TRAT1				

## 4 Discussion

Our data combines multiple elements that may be involved in the onset of acute pain that may be the drivers of pain chronification. In contrast to prior reports that have focused on nerve damage using MRI metrics of nerve damage (viz., DTI) we provide data relating to multidimensional neurobiological and psychophysical processes that occur subsequent to a common non-surgical condition producing nerve damage. In a cohort of adolescent patients, who had suffered an acute ankle injury, we evaluated clinical/neurological, psychological, nerve and brain metrics. Our findings indicated that within 3 months of an acute ankle injury, significant changes are noted in the peripheral nerve (as measured by DTI); an ongoing inflammatory process (as measured by miRNA) and functional and structural brain changes (as measured by resting state fMRI, gray matter morphology, and white matter integrity (DTI)). Taken together the data provides insights into a multifactorial paradigm of how pain and inflammation may drive changes in the peripheral and central nervous system that can be quantitatively assessed.

### 4.1 Ankle injury as a harbinger of neuropathic pain

All patients had a history of ankle injury within 3 months of being evaluated. Elements of neuropathic pain were observed in the affected ankle in all patients (viz., hypersensitivity or hyposensitivity to mechanical (pin, cold) stimuli; summation, or allodynia). Peak incidence of ankle injury occurs among teenagers in an approximately equal male:female ratio (Waterman et al., 2010). Following ankle injury, injury to both the tibial and fibular nerve have been reported with estimates of the latter occurring in up to 80% of cases (Hunt, 2003). While most recover in the months following the injury, estimates for pain chronicity may occur in up to 40% (Safran et al., 1999) of individuals. Thus, this injury provides an ideal model to evaluate (1) the evolution/devolution of neuropathic pain, and (2) neurobiological (brain and nerve), immunological and psychological changes that occur over time in a “natural injury”.

### 4.2 Measures of nerve fiber integrity

Currently, objective measures of nerve fiber changes include EMG for large myelinated fibers (Lazaro, 2015) and single fiber measures for unmyelinated fibers (Jonas et al., 2018, Serra, 2012). Recent work has indicated that measures of whole nerve fiber in humans can provide another measure of nerve damage based on diffusion tensor imaging (DeSouza et al., 2014, Hung et al., 2017, Vaeggemose et al., 2017a, Vaeggemose et al., 2017b). As noted, our measures include a region of interest analysis that captures the Sciatic nerve above and below at its’ bifurcation into the two major nerves that go on to innervate the ankle joint. These are the fibular and tibial nerves innervating the lateral and medial aspects of the ankle and can be damaged by eversion (stretching the fibial n.) and inversion (stretching the tibial n.) injury. The approach adopted in this investigation was sensitive to both tibial and fibular injury and included only persons with unilateral ankle injury. Pure lateral and medial contributions from an ankle injury are probably unlikely and hence measures of the opposite nerves have provided controls for this effect.

Findings from the peripheral nerve analysis showed significant interactions for FA, RD, MD and AD but not for the T2-weighted signal. Post-hoc analyses show that changes in DWI-metrics are observed in all divisions (Sciatic, Tibial, Fibular) of the evaluated nerve sample. A decrease in AD in the tibial and sciatic nerve division (see Fig. 2) were observed in our cohort and is consistent with models of axonal degeneration showing that a decrease in AD reflects a decrease in axonal integrity (Sun et al., 2008). Alternatively, radial diffusivity is typically associated with changes in myelination where demyelination would result in an increase in radial diffusivity (Song et al., 2003). This aligns with the current cohort where an increase in RD was found in the sciatic nerve, suggesting a less restrictive environmental relative to healthy controls and the potential for demyelination. Notably, in rat models, a decrease in FA and increase in RD have been observed during the degenerative phases and the inverse was observed during the regenerative phases following peripheral nerve injury (Yamasaki et al., 2015). Taken in combination with an increase in FA found within the fibular nerve, these findings suggest that persons in the current ankle injury cohort may be at different stages ranging between degeneration and remodeling. Group level effects in T2-weighted signal and mean diffusivity from the sciatic nerve suggest the presence of an inflammatory component in the ankle injury cohort (Alexander et al., 2008). Mean diffusivity has been shown to be increased after peripheral nerve injury and subsequently return to baseline (Chen et al., 2017) and its variable nature may also address inconsistent findings in other diffusion tensor metrics (Winklewski et al., 2018). Thus, state-of-the-art high-resolution DTI allowed for (1) the visual identification and evaluation of separate fiber bundles of the tibial and fibular nerve after the bifurcation of the sciatic nerve and (2) identification of atypical diffusivity in our neuropathic pain cohort suggesting the presence of an abnormal structure in the nerve that may reflect ongoing neuropathology and inflammation and may be used as a predictor of treatment response. Using nerve fiber data from both legs allowed for a more precise evaluation of determination that nerve structure integrity is impacted in persons in the ankle injury cohort.

### 4.3 Inflammatory response

The inflammatory response to peripheral nerve damage was highly significant in our ankle injury cohort (see Fig. 3). Notably, in response to peripheral nerve injury, the canonical response is an activation/proliferation of resident inflammatory cells, immune cell recruitment/migration, phagocytosis and finally resolution of inflammation occurs (Ellis and Bennett, 2013, Moalem and Tracey, 2006). This response naturally integrates mast cells, cytokines (e.g., IL-1B), T-cells, and other microglia amongst other cells that have the capacity to infiltrate the central nervous system (Ellis and Bennett, 2013, Gu et al., 2016, Peng et al., 2016). Inflammatory molecules including cytokines may not only contribute as activators and in the maintenance of pain in the peripheral and activate central (spinal cord and brain) but also contribute to depression like symptoms as reported in preclinical models (Gui et al., 2016). It should be noted that some cytokines are protective or restorative (e.g., IL-10) (Siqueira Mietto et al., 2015).

The traditional inflammatory response appears to be altered in our ankle injury cohort as a prolonged upregulation of distinct pathways is present. Gene expression was focused on five principle pathways that govern the bodies response to peripheral nerve injury (see Fig. 4). Elevated expression in the neuroinflammation signaling pathway and the glucocorticoid signaling pathway point towards a duality in elevated pro-inflammatory cytokines and inflammatory molecules (NFP), but also attempts to suppress this activity (GSP). This is consistent with evidence of axonal degeneration found in our ankle injury cohort and suggests that a diffuse inflammatory process is persisting in our neuropathic pain group. That is, the GSP is associated with anti-inflammatory properties, and delayed wound healing, and within the nervous system is associated with physiological homeostasis and responses to stressors. Elevated levels found in this cohort agree with prior work examining the response to peripheral nerve damage (Tung et al., 2015) and interactive network analyses demonstrate the extent to which elevations in these pathways may be disseminated.

### 4.4 Changes in gray matter volume

Our data showed significant decreases were found in the pre- and post-central gyri of the left and right hemispheres in the somatosensory cortex in regions that approximated the sensory and motor representations of the injured foot. Observed decreases in cortical thickness may be the product of pain from the peripheral nerve injury or the specific disuse of the region that could lead to somatotopic changes. With respect to pain, chronic pain produces changes in gray matter (GM) volume that are thought to reflect changes in dendritic complexity (Borsook et al., 2013, Langer et al., 2012), decreased glial volume, or decreased regional vasculature (Langer et al., 2012). In the somatosensory cortex, both increases (e.g., in episodic migraine (Kim et al., 2015)) and decreases (e.g., trigeminal neuralgia (Obermann et al., 2013)) have been reported.

In the current cohort, we extracted correlations between measures of physical activity/disability through the FDI and correlated them against cortical thickness in the pre- and postcentral gyrus. No significant correlations were found (postcentral gyrus: r < 0.25, p > 0.32; precentral gyrus: r < 0.06, p > 0.083). This may be a product however of integrating both left and right ankle injury subjects as decreased grey matter is reported in the contralateral brain hemisphere in individuals with motor-sensory loss (i.e., limb amputation (Jiang et al., 2015)). To this point, we performed a follow-up investigation comparing participants with left, versus right sided injuries. As shown in Supplementary Table 2 group differences were observed within the precentral gyrus, suggesting a contralateral correspondence between decreased thickness and side of injury (i.e., left ankle injury – right hemisphere decrease). Although some brain regions appeared to be asymmetrically impacted such as the rostral middle frontal gyrus, precuneus and caudal anterior cingulate, a number of regions were symmetrically impacted in the ankle injury cohort. Alternatively, a number of brain regions showed evidence of increases in cortical thickness in the ankle injury cohort. As outlined previously (Simons, 2016), cortical thickness has been observed to increase in regions associated with pain processing in patient groups. In line with this, Supplementary Table 3 highlights two important observations. First, regions found to correlate with psychometrics (PPST, FOPQ, and PCS) were external to the pre- and postcentral gyrus where we observed decreased thickness in the ankle sprain cohort. Second, a number of brain regions that show a positive correlation with symptom reporting are also reported as being thicker in the ankle sprain cohort relative to healthy controls (e.g., lateral orbitofrontal, pars opercularis, inferior temporal). As such, findings may allude to independent, yet inter-related, processes occurring wherein functional dis-use could lead to decreases in cortical thickness and corresponding pain to increases in cortical thickness.

### 4.5 Changes in white matter integrity

We observed using significant group differences in mean diffusivity in five of the eighteen tracts (see Section 3). Mean diffusivity reflects changes in the cellular membrane (Bosch et al., 2012) and may signal fluid accumulation consistent with inflammation (Alexander et al., 2008). Only minimal changes were observed in FA that approached significance in the SPL that also showed changes in axial and radial diffusivity. The superior longitudinal fasciculus connects the temporoparietal areas with frontal areas with projections to the precentral gyrus (Bernal and Altman, 2010) and aligns with grey matter observations from the temporal and frontal lobes. The pathways showing changes in mean diffusivity were SPL (temporal and parietal), IFL, and ATR which have all shown a common thread with integration of sensory information (Herbet et al., 2018) and processing of pain (ATR). The underlying mechanism of these changes in white matter may relate to ongoing afferent barrage of nociceptor drive from the periphery (Thacker et al., 2007) and or inflammatory processes that contribute to glial changes supporting these white matter connections. Together, results can be interpreted to suggest that a diffuse form of brain injury is present in this cohort that includes effects on the white matter pathway (SFL) specifically implicated in processing of sensory-motor information.

### 4.6 Functional brain changes

While the human connectome is a complex system, there are some regions of the brain that are salient in pain processing. Using resting state measures we found significant changes in functional connectivity (Fc) in three areas used as ROI’s known to be involved in pain processing: (1) the Nucleus Accumbens – as it relates to aversive evaluation (Klawonn and Malenka, 2019, Wulff et al., 2018); (2) the Amygdala – as it relates to fear of pain or anxiety (Neugebauer, 2015); and the Periaqueductal gray (Heinricher, 2016, Linnman et al., 2012, Ren and Dubner, 2002) – as it relates to pain modulation. We also found group differences in how functional networks are structured in the ankle injury group where the inferior temporal cortex was found to engage near-by brain regions more so than in healthy controls.

Observed differences in a priori pain-related ROIs support a decrease in functional connectivity in the ankle injury relative to the healthy control group. Prior investigations in pain populations have shown evidence to support a decrease in functional connectivity between pain regions of the brain (Androulakis et al., 2018, Gao et al., 2016, Liu et al., 2018). Alternatively, it is interesting to note that connectivity with the amygdala approached significance in our pain cohort, aligning with the increased reporting of pain symptoms and fear of pain. The use of graph theory analysis allowed for a unique perspective regarding network structure changes in persons with neuropathic pain. Findings of changes in local efficiency and clustering coefficient suggest that the inferior temporal gyrus (left hemisphere) had increased connectivity with surrounding nodes relative to healthy controls. Prior research using different models of neuropathy has shown evidence of increased resting state network connectivity of the inferior temporal gyrus within the default mode network (Cauda et al., 2009) as well as evidence to suggest gray matter volume within this area of the cortex is negatively correlated with pain intensity and disease duration (Wang et al., 2017) as well as encoding of the pain experience (Lee et al., 2017). As such, findings from the functional connectivity analysis provide support for altered functional processing in pain-related ROIs as well as changes in network structure that may earmark the preliminary stages of pain chronification.

### 4.7 Behavioral changes in response to peripheral nerve damage

Median scores for the ankle injury cohort in the current investigation fell into the low level of fear of pain; however, participants in the ankle injury cohort ranged into the high fear of pain level. For pain catastrophizing, both groups had median levels that reflected mild levels of pain catastrophizing with the patient group extending into the severe catastrophizing level for the ankle injury cohort (healthy controls did not exceed past the mild level). Both groups showed minimal levels of disability as assessed using the functional disability scale. Interestingly, the above findings support the behavioral impact that even when in the acute setting, patient with neuropathic pain experience elevated levels of pain catastrophizing and fear of pain from their injuries (Elman and Borsook, 2018, Simons, 2016, Vlaeyen and Linton, 2000). Ongoing pain may produce changes in cognitive and emotional function. Such changes may exacerbate the current phenotype viz., individuals with catastrophizing may have an exaggerated symptomatic response as is the case with preoperative condition (Theunissen et al., 2012) and brain response (Gracely, 2004) to their pain. Negative affect may be induced by the ankle trauma. Patients with neuropathic pain frequently report higher levels on self-report measures of fear of pain, pain catastrophizing and pain screening tools that are associated with pain intensity, pain disability and self-efficacy (McCahon et al., 2005). This finding is thought to be associated with fear conditioning and abnormal brain activity in regions such as the amygdala (see above) as well as other brain regions such as the insula and anterior cingulate cortex (Simons, 2016). In addition to pain/nociceptive drive, immune responses may drive alterations in affective feelings (e.g., depression) which may contribute to pain processing at a central level (Leonard, 2010) independent of inflammatory changes driving an increased sensitization in peripheral and central (sensory, emotional, cognitive, modulatory) pain pathways.

### 4.8 A cascade of processes

**Fig. 7:**
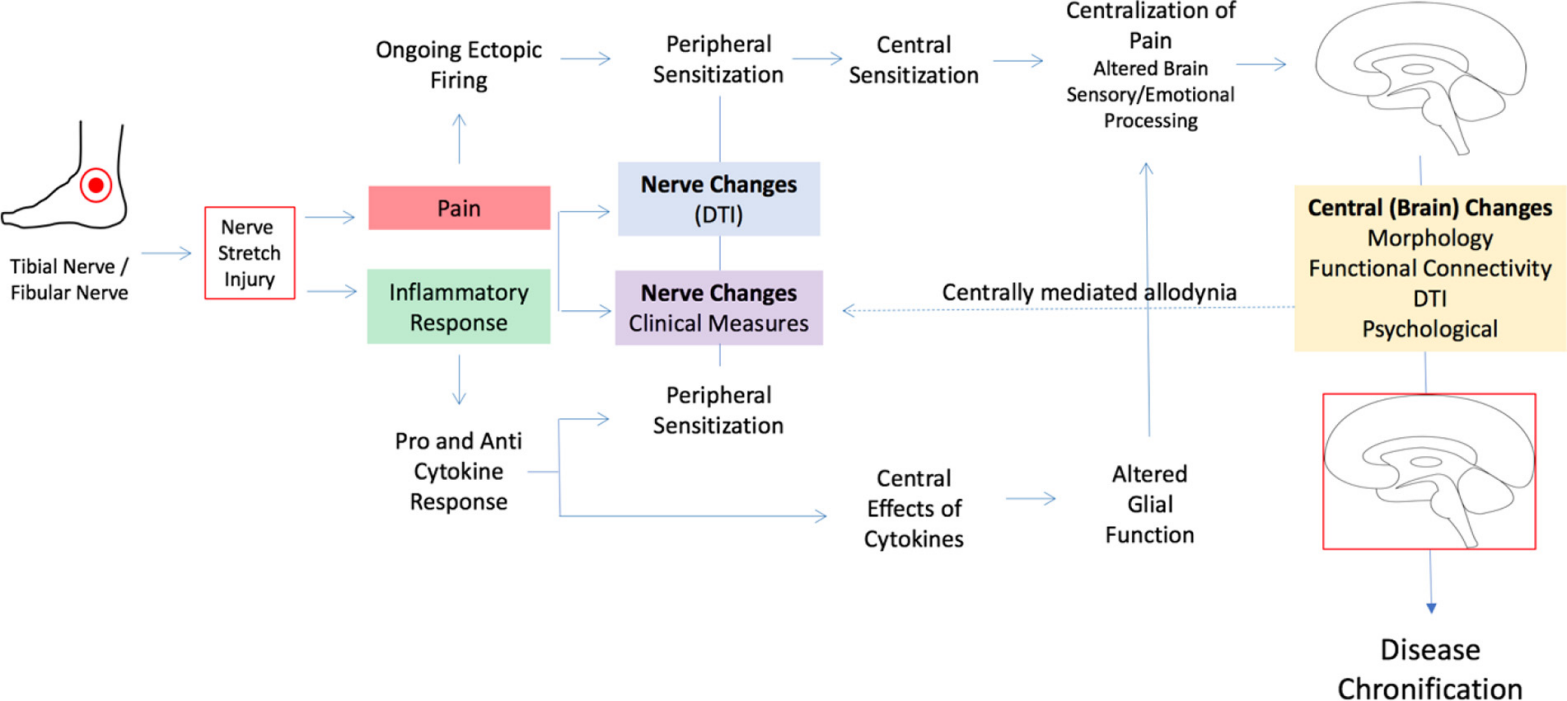
Proposed cascade model of nerve stretch injury. Results from this investigation are integrated into a proposed model to understand how acute findings may relate to chronic pain processes.

Following a nerve injury, it is unclear why some individuals go on to have chronic pain and others recover. As noted in this study, following what could be a relatively trivial injury, a cascade of processes unfold that occur within the peripheral and central nervous system. The unfolding of abnormal nerve activity shown by single fiber neurography in nerved damaged patients provides a model that is likely to be present in the patients evaluated. This together with inflammatory processes that align with observations of peripheral sensitization (Moalem and Tracey, 2006, Thacker et al., 2007), provides a driver for an afferent barrage that may alter the function and structure of the central nervous system. These observations are supported when viewing the data using a cross-correlation analysis (see Fig. 6). Unique relationships showing strong positive correlations are observed in the ankle sprain cohort relative to healthy controls in genetics (compare section A′ against A″) and brain imaging (compare B′ against B″) which may underlie changes in inflammation and brain regions such as the somatosensory system, respectively. It can also be appreciated how some domains may interact such as in the case of genetic and imaging markers that display a unique pattern in each group (Fig. 6 – Section A–B), supporting how inflammatory molecules may influence brain health. This is supported by extracted features that show how inflammatory molecules relating to cell cycle and the stress response (Rosati et al., 2011) as well as brain regions implicated in sensory integration (superior longitudinal fasciculus (Herbet et al., 2018)) and the adaptation to chronic stressors such as pain (Caudate (Wunderlich et al., 2011)) were the highest correlated in the ankle injury cohort. Moreover, recently published work from our lab has shown that regions such as the inferior temporal gyrus and orbitofrontal cortex had a similar increase in acute neuropathic pain patients (Youssef et al., 2019). These same regions were found to further increase in chronic pain patients suggesting a potential for regions identified in this investigation to be implicated in the development of chronic pain. These changes likely morph an acute pain process to a chronic pain process as described by others (Moalem and Tracey, 2006). Of note are the changes in white and gray matter in regions that initially mostly affect somatosensory systems, but also embrace neural nodes or regions involved in pivotal processes in pain chronification. Such changes may contribute to a feed forward loop that contributes to the chronification process which we have outlined in Fig. 7. The canonical response of inflammation and nociception can instigate, through aberrant control of pro and anti-inflammatory molecules as well as progressive neurological changes, the centralization of pain and disease chronification.

## 5 Limitations

There are a number of limitations that include: (1) The investigation was a cross-section evaluation of neuropathic pain, and as such we cannot comment on the longitudinal progression of the condition and the causal nature of any domain. (2) Our sample size for healthy controls was relatively low and defined initially based on the analyses methods of the genomics portion of the investigation. This, along with the patient cohort limited our ability to perform feature reduction or classification methods across domains (3) Where possible multiple-comparison corrections were performed; however, based on the restricted sample size and pre-defined comparisons in select analyses (e.g., psychological data) we elected to use the cutoff of p < 0.05. (4) Other biomarkers such as skin biopsy for epidermal nerve fiber density were not performed in this cohort since we did not wish to do this procedure in healthy teenagers. (5) Given that we were not able to evaluate patients prior to their injury, we compared patients with healthy controls; however, issues such a baseline psychological or inflammatory levels were therefore not possible (6) Patients who had either left or right ankle involvement were included in the study. When comparing the patient groups to each other based on a right or left ankle injury, findings were largely symmetrical (see Supplementary details) suggesting that the greater number of left ankle injury subjects in this investigation did not impact study findings.

## 6 Conclusions

Understanding the mechanisms through which peripheral nerve damage results in long-term chronic pain would allow improved diagnostics and patient monitoring. In this investigation, we take several steps forward by quantifying the extent of nerve pathology and showing how this corresponds with a diffuse amplification of inflammatory activity. Our data provide support for a complex process unfolding following nerve injury. The integrative approach of clinical and psychological measures with imaging (brain and nerve) and genetic markers of inflammation provide a window into the initiation of a cascade of events that may drive towards the pathological state of chronic pain. Clearly, these changes are immediate and challenge the notion of a time-related point of “when pain becomes chronic”. Nerve injury from ankle injury produces early changes in nerve and inflammatory markers that may drive central changes in the brain. Brain changes are likely the combined result of secondary atrophic events in combination with direct inflammatory and psychological changes. The data provide a model to evaluate a feed forward process as well as potential restorative processes in such patients, where measures of these same systems (nerve, inflammation, brain, neurological and psychological variables) may be time locked as predictors of potential recovery or treatment efficacy. For example, the resolution or expression of proinflammatory cytokines may be the harbinger of diminished neural afferent drive, affecting as well, central sensory, emotional cognitive and modulatory processes. Future work is aimed at understanding the evolution and potential devolution of neuropathic pain to shed light on the individual contributions of each domain to chronic neuropathic pain.

## Declaration of Competing Interest

The authors declare that they have no known competing financial interests or personal relationships that could have appeared to influence the work reported in this paper.

## References

[b0005] Alexander A.L., Lee J.E., Lazar M., Field A.S. (2008). Diffusion tensor imaging of the brain. Neurotherapeutics.

[b0010] Androulakis X.M., Krebs K.A., Jenkins C., Maleki N., Finkel A.G., Rorden C., Newman R. (2018). Central executive and default mode network intranetwork functional connectivity patterns in chronic migraine. J. Neurol. Disord..

[b0015] Angin M., Sharma S., King M., Murooka T.T., Ghebremichael M., Mempel T.R., Addo M.M. (2014). HIV-1 infection impairs regulatory T-cell suppressive capacity on a per-cell basis. J. Infect. Dis..

[b0020] Apkarian A.V., Reckziegel D. (2018). Peripheral and central viewpoints of chronic pain, and translational implications. Neuroscience Letters.

[b0025] Baron R., Maier C., Attal N., Binder A., Bouhassira D., Cruccu G., Treede R.-D. (2017). Peripheral neuropathic pain: a mechanism-related organizing principle based on sensory profiles. Pain.

[b0030] Bernal B., Altman N. (2010). The connectivity of the superior longitudinal fasciculus: a tractography DTI study. Magnetic Resonance Imaging.

[b0035] Borsook D., Erpelding N., Becerra L. (2013). Losses and gains: Chronic pain adn altered brain morphology. Expert Rev. Neurother..

[b0040] Borsook D., Youssef A.M., Simons L., Elman I., Eccleston C. (2018). When pain gets stuck: The evolution of pain chronification and treatment resistance. Pain.

[b0045] Bosch B., Arenaza-Urquijo E.M., Rami L., Sala-Llonch R., Junqué C., Solé-Padullés C., Bartrés-Faz D. (2012). Multiple DTI index analysis in normal aging, amnestic MCI and AD. Relationship with neuropsychological performance. Neurobiology of Aging.

[b0050] Cauda F., Sacco K., Duca S., Cocito D., D’Agata F., Geminiani G.C., Canavero S. (2009). Altered resting state in diabetic neuropathic pain. PLoS ONE.

[b0055] Chen Y.-Y., Zhang X., Lin X.-F., Zhang F., Duan X.-H., Zheng C.-S., Shen J. (2017). DTI metrics can be used as biomarkers to determine the therapeutic effect of stem cells in acute peripheral nerve injury: DTI of stem cell therapy in nerve injury. Journal of Magnetic Resonance Imaging.

[b0060] Colloca L., Ludman T., Bouhassira D., Baron R., Dickenson A.H., Yarnitsky D., Raja S.N. (2017). Neuropathic pain. Nature Reviews Disease Primers.

[b0065] DeSouza D.D., Hodaie M., Davis K.D. (2014). Abnormal trigeminal nerve microstructure and brain white matter in idiopathic trigeminal neuralgia. Pain.

[b0070] Leonard E. (2010). The concept of depression as a dysfunction of the immune system. Current Immunology Reviews.

[b0075] Ellis A., Bennett D.L.H. (2013). Neuroinflammation and the generation of neuropathic pain. British Journal of Anaesthesia.

[b0080] Elman I., Borsook D. (2018). Threat response system: parallel brain processes in pain vis-à-vis fear and anxiety. Frontiers in Psychiatry.

[b0085] Fischl B., Dale A.M. (2000). Measuring the thickness of the human cerebral cortex from magnetic resonance images. Proc. Natl. Acad. Sci. U.S.A..

[b0090] Gao Q., Xu F., Jiang C., Chen Z., Chen H., Liao H., Zhao L. (2016). Decreased functional connectivity density in pain-related brain regions of female migraine patients without aura. Brain Research.

[b0095] Giuffre B.A., Jeanmonod R. (2018). Anatomy, Sciatic Nerve.

[b0100] Gracely R.H. (2004). Pain catastrophizing and neural responses to pain among persons with fibromyalgia. Brain.

[b0105] Gu N., Peng J., Murugan M., Wang X., Eyo U.B., Sun D., Wu L.-J. (2016). Spinal microgliosis due to resident microglial proliferation is required for pain hypersensitivity after peripheral nerve injury. Cell Rep..

[b0110] Gui W.-S., Wei X., Mai C.-L., Murugan M., Wu L.-J., Xin W.-J., Liu X.-G. (2016). Interleukin-1β overproduction is a common cause for neuropathic pain, memory deficit, and depression following peripheral nerve injury in rodents. Mol. Pain.

[b0115] Hashmi J.A., Baliki M.N., Huang L., Baria A.T., Torbey S., Hermann K.M., Apkarian A.V. (2013). Shape shifting pain: chronification of back pain shifts brain representation from nociceptive to emotional circuits. Brain.

[b0120] Heinricher M. (2016). Pain modulation and the transition from acute to chronic pain.

[b0125] Hemington K.S., Wu Q., Kucyi A., Inman R.D., Davis K.D. (2016). Abnormal cross-network functional connectivity in chronic pain and its association with clinical symptoms. Brain Structure & Function.

[b0130] Herbet G., Zemmoura I., Duffau H. (2018). Functional anatomy of the inferior longitudinal fasciculus: from historical reports to current hypotheses. Frontiers in Neuroanatomy.

[b0135] Hubbard C.S., Becerra L., Smith J.H., Delange J.M., Smith R.M., Black D.F. (2016). Brain changes in responders vs. non-responders in chronic migraine: markers of disease reversal. Front. Human Neurosci..

[b0140] Hung P.S.-P., Chen D.Q., Davis K.D., Zhong J., Hodaie M. (2017). Predicting pain relief: use of pre-surgical trigeminal nerve diffusion metrics in trigeminal neuralgia. NeuroImage: Clin..

[b0145] Hunt G.C. (2003). Injuries of peripheral nerves of the leg, foot and ankle: an often unrecognized consequence of ankle sprains. Foot.

[b0150] Ji R.-R., Nackley A., Huh Y., Terrando N., Maixner W. (2018). Neuroinflammation and central sensitization in chronic and widespread pain. Anesthesiology.

[b0155] Jiang G., Yin X., Li C., Li L., Zhao L., Evans A.C., Wang J. (2015). The plasticity of brain gray matter and white matter following lower limb amputation. Neural Plast..

[b0160] Jonas R., Namer B., Stockinger L., Chisholm K., Schnakenberg M., Landmann G., Rukwied R. (2018). Tuning in C-nociceptors to reveal mechanisms in chronic neuropathic pain: C-nociceptor activation by sinusoidal stimulation. Annals of Neurology.

[b0165] Kim E.J., Pellman B., Kim J.J. (2015). Stress effects on the hippocampus: a critical review. Learn. Mem..

[b0170] Klawonn A.M., Malenka R.C. (2019). Nucleus accumbens modulation in reward and aversion. Cold Spring Harbor Symposia on Quantitative Biology.

[b0175] Kovacs M. (2010). Children’s Depression Inventory 2.

[b0180] Langer N., Hanggi J., Muller N.A., Simmen H.P., Jancke L. (2012). Effects of limb immobilization on brain plasticity. Neurology.

[b0185] Lazaro R.P. (2015). Electromyography in musculoskeletal pain: a reappraisal and practical considerations. Surg. Neurol. Int..

[b0190] Lee P.-S., Low I., Chen Y.-S., Tu C.-H., Chao H.-T., Hsieh J.-C., Chen L.-F. (2017). Encoding of menstrual pain experience with theta oscillations in women with primary dysmenorrhea. Scientific Reports.

[b0195] Linnman C., Moulton E.A., Barmettler G., Becerra L., Borsook D. (2012). Neuroimaging of the periaqueductal gray: state of the field. NeuroImage.

[b0200] Littlejohn G. (2015). Neurogenic neuroinflammation in fibromyalgia and complex regional pain syndrome. Nature Reviews Rheumatology.

[b0205] Liu J., Zhang F., Liu X., Zhuo Z., Wei J., Du M., Wang D. (2018). Altered small-world, functional brain networks in patients with lower back pain. Sci. China Life Sci..

[b0210] March J.S., Parker J.D.A., Sullivan K., Stallings P., Conners C. (2013). The multidimensional anxiety scale for children (MASC): factor structure, reliability, and validity. Journal of the American Academy of Child and Adolescent Psychiatry.

[b0215] McCahon S., Strong J., Sharry R., Cramond T. (2005). Self-report and pain behavior among patients with chronic pain. Clin. J. Pain.

[b0220] Menorca R.M.G., Fussell T.S., Elfar J.C. (2013). Nerve physiology. Hand Clinics.

[b0225] Moalem G., Tracey D.J. (2006). Immune and inflammatory mechanisms in neuropathic pain. Brain Research Reviews.

[b0230] Neugebauer V. (2015). Amygdala pain mechanisms.

[b0235] Obermann M., Rodriguez-Raecke R., Naegel S., Holle D., Mueller D., Yoon M.-S., Katsarava Z. (2013). Gray matter volume reduction reflects chronic pain in trigeminal neuralgia. NeuroImage.

[b0240] Ogilvie-Harris D.J., Gilbart M.K., Chorney K. (1997). Chronic pain following ankle sprains in athletes: the role of arthroscopic surgery. Arthroscopy.

[b0245] Peng J., Gu N., Zhou L., Eyo U., Murugan M., Gan W.-B., Wu L.-J. (2016). Microglia and monocytes synergistically promote the transition from acute to chronic pain after nerve injury. Nature Communications.

[b0250] Perry C.J., Blake P., Buettner C., Papavassiliou E., Schain A.J., Bhasin M.K., Burstein R. (2016). Upregulation of inflammatory gene transcripts in periosteum of chronic migraineurs: Implications for extracranial origin of headache. Annals of Neurology.

[b0255] Peterson A.C., Crockett L., Richards M., Boxer A. (1988). A self-report measure of pubertal status: reliability, validity, and initial norms. J. Youth Adolecs..

[b0260] R Core Team (2019). R: A Language and Environment for Statistical Computing. https://www.R-project.org.

[b0265] Ren K., Dubner R. (2002). Descending modulation in persistent pain: an update. Pain.

[b0270] Ritchie M.E., Phipson B., Wu D., Hu Y., Law C.W., Shi W., Smyth G.K. (2015). Limma powers differential expression analyses for RNA-sequencing and microarray studies. Nucleic Acids Res..

[b0275] Rosati A., Graziano V., De Laurenzi V., Pascale M., Turco M.C. (2011). BAG3: a multifaceted protein that regulates major cell pathways. Cell Death and Disease.

[b0280] Safran M.R., Benedetti R.S., Bartologzzi A.R., Mandelbaum B.R. (1999). Lateral ankle sprains: a comprehensive review Part 1: Etiology, pathoanatomy, histopahogenesis, and diagnosis. Medicine and Science in Sports and Exercise.

[b0285] Schafers M., Lee D.H., Brors D., Yaksh T.L., Sorkin L.S. (n.d.). Increased sensitivity of injured and adjacent uninjured rat primary sensory neurons to exogenous tumor necrosis factor-after spinal nerve ligation. 11. 10.1523/JNEUROSCI.23-07-03028.2003 PMC6742101 12684490

[b0290] Serra J. (2012). Microneurography: towards a biomarker of spontaneous pain. Pain.

[b0295] Simons L.E. (2016). Fear of pain in children and adolescents with neuropathic pain and complex regional pain syndrome. Pain.

[b0300] Simons L.E., Smith A., Ibagon C., Coakley R., Logan D.E., Schechter N., Hill J.C. (2015). Pediatric pain screening tool: rapid identification of risk in youth with pain complaints. Pain.

[b0305] Simons L.E., Sieberg C.B., Carpino E., Logan D., Berde C. (2011). The fear of pain questionnaire (FOPQ): assessment of pain-related fear among children and adolescents with chornic pain. J. Pain.

[b9000] Simons L.E., Erpelding N., Hernandez J.M., Serrano P., Zhang K., Lebel A., Sethna N., Berde C., Prabhu S.P., Becerra L., Borsook D. (2016). Fear and reward circuit alterations in pediatric CRPS. Front. Human Neurosci..

[b0310] Siqueira Mietto B., Kroner A., Girolami E.I., Santos-Nogueira E., Zhang J., David S. (2015). Role of IL-10 in resolution of inflammation and functional recovery after peripheral nerve injury. Journal of Neuroscience.

[b0315] Song S.-K., Sun S.-W., Ju W.-K., Lin S.-J., Cross A.H., Neufeld A.H. (2003). Diffusion tensor imaging detects and differentiates axon and myelin degeneration in mouse optic nerve after retinal ischemia. NeuroImage.

[b0320] Sullivan M. (1995). The Pain Catastrophizing Scale.

[b0325] Sun Q., Tu H., Xing G.-G., Han J.-S., Wan Y. (2005). Ectopic discharges from injured nerve fibers are highly correlated with tactile allodynia only in early, but not late, stage in rats with spinal nerve ligation. Experimental Neurology.

[b0330] Sun S.-W., Liang H.-F., Cross A.H., Song S.-K. (2008). Evolving Wallerian degeneration after transient retinal ischemia in mice characterized by diffusion tensor imaging. NeuroImage.

[b0335] Thacker M.A., Clark A.K., Marchand F., McMahon S.B. (2007). Pathophysiology of peripheral neuropathic pain: immune cells and molecules. Anesthesia and Analgesia.

[b0340] Theunissen M., Peters M.L., Bruce J., Gramke H.-F., Marcus M.A. (2012). Preoperative anxiety and catastrophizing: a systematic review and meta-analysis of the association with chronic postsurgical pain. Clin. J. Pain.

[b0345] Tracey I., Woolf C.J., Andrews N.A. (2019). Composite pain biomarker signatures for objective assessment and effective treatment. Neuron.

[b0350] Tung K.-W., Behera D., Biswal S. (2015). Neuropathic pain mechanisms and imaging. Semin. Musculoskel. Radiol..

[b0355] Vaeggemose M., Pham M., Ringgaard S., Tankisi H., Ejskjaer N., Heiland S., Andersen H. (2017). Magnetic resonance neurography visualizes abnormalities in sciatic and tibial nerves in patients with type 1 diabetes and neuropathy. Diabetes.

[b0360] Vaeggemose M., Vaeth S., Pham M., Ringgaard S., Jensen U.B., Tankisi H., Andersen H. (2017). Magnetic resonance neurography and diffusion tensor imaging of the peripheral nerves in patients with Charcot-Marie-Tooth Type 1A: DTI of peripheral nerves in CMT1A. Muscle and Nerve.

[b0365] van Ochten J.M., van Middelkoop M., Meuffels D., Bierma-Zeinstra S.M.A. (2014). Chronic complaints after ankle sprains: a systematic review on effectiveness of treatments. Journal of Orthopaedic and Sports Physical Therapy.

[b0370] Vlaeyen J.W.S., Linton S.J. (2000). Fear-avoidance and its consequences in chronic musculoskeletal pain: a state of the art. Pain.

[b0375] Walker L.S., Greene J.W. (1991). The Functional Disability Inventory (FDI): Measuring a neglected dimension of child health status. Journal of Pediatric Psychology.

[b0380] Wang Y., Cao D., Remeniuk B., Krimmel S., Seminowicz D.A., Zhang M. (2017). Altered brain structure and function associated with sensory and affective components of classic trigeminal neuralgia. Pain.

[b0385] Waterman C.B.R., Owens M.B.D., Davey C.S., Zacchilli C.M.A., Belmont L.C.P.J. (2010). The epidemiology of ankle sprains in the United States. J. Bone Joint Surg. Am.

[b0390] Whitfield-Gabrieli S., Nieto-Castanon A. (2012). Conn: a functional connectivity toolbox for correlated and anticorrelated brain networks. Brain Connectivity.

[b0395] Winklewski P.J., Sabisz A., Naumczyk P., Jodzio K., Szurowska E., Szarmach A. (2018). Understanding the physiopathology behind axial and radial diffusivity changes—what do we know?. Frontiers in Neurology.

[b0400] Woolf C.J. (2011). Central sensitization: implications for the diagnosis and treatment of pain. Pain.

[b0405] Wulff A.B., Tooley J., Marconi L.J., Creed M.C. (2018). Ventral pallidal modulation of aversion processing. Brain Research.

[b0410] Wunderlich A.P., Klug R., Stuber G., Landwehrmeyer B., Weber F., Freund W. (2011). Caudate nucleus and insular activation during a pain suppression paradigm comparing thermal and electrical stimulation. Open Neuroimag. J..

[b0415] Yamasaki T., Fujiwara H., Oda R., Mikami Y., Ikeda T., Nagae M., Kubo T. (2015). In vivo evaluation of rabbit sciatic nerve regeneration with diffusion tensor imaging (DTI): correlations with histology and behavior. Magnetic Resonance Imaging.

[b0420] Youssef A.M., Azqueta-Gavaldon M., Silva K.E., Barakat N., Lopez N., Mahmud F., Borsook D. (2019). Shifting brain circuits in pain chronicity. Human Brain Mapping.

