# Supplementary material for: Biological and behavioral markers of pain following nerve injury in humans
Source: Neurobiol Pain. 2019 Dec 4;7:100038. doi: 10.1016/j.ynpai.2019.100038 (PMC6926375; doi:10.1016/j.ynpai.2019.100038)
Supplement: Supplementary data 2 [file mmc2.docx]

| Left Hemisphere | | x | y | z | size (mm^2^) | Right Hemisphere | | x | y | z | size (mm^2^) |
| --- | --- | --- | --- | --- | --- | --- | --- | --- | --- | --- | --- |
| Right Injury > Left Injury | |  |  |  |  | Right Injury > Left Injury | |  |  |  |  |
|  | *Precuneus* | -7.1 | -62.4 | 41.3 | 46.01 |  | *Inferior parietal* | 32.3 | -56 | 40.8 | 78.19 |
|  | *Caudal anterior cingulate* | -6.6 | 20.1 | 28.3 | 34.42 |  | *Fusiform* | 35.7 | -14.3 | -33 | 41.43 |
|  | *Precentral* | -26.2 | -14 | 57.6 | 33.54 |  | *Middle temporal* | 56.3 | -51.4 | -3.5 | 24.18 |
|  | *Fusiform* | -34.5 | -7.9 | -36.4 | 44.14 |  | *Superior frontal* | 16.1 | 31 | 51.7 | 25.4 |
|  | *Inferior parietal* | -41.2 | -74.7 | 27.9 | 9.62 |  | *Precuneus* | 10.5 | -48.6 | 43.5 | 13.18 |
|  | *Superior frontal* | -17.5 | 7.9 | 62.5 | 36.45 |  | *Superior frontal* | 8.1 | 41.6 | 28.1 | 15.94 |
|  | *Precentral* | -36.8 | -2.6 | 43.2 | 55.48 |  | *Superior parietal* | 33.5 | -50.8 | 60.4 | 5.7 |
|  | *Rostral middle frontal* | -27.5 | 29.5 | 35.5 | 8.27 |  | *Paracentral* | 14.2 | -22 | 45.9 | 2.5 |
|  | *Precentral* | -36.4 | -10.6 | 59.3 | 15.93 |  | *Superior parietal* | 14 | -65.5 | 57.2 | 9.19 |
|  | *Supramarginal* | -53.9 | -53.2 | 22.6 | 11.51 |  | *Middle temporal* | 53.8 | -28.7 | -18.2 | 8.86 |
|  |  |  |  |  |  |  | *Supramarginal* | 57.3 | -42.1 | 26.4 | 3.53 |
|  |  |  |  |  |  |  | *Superior parietal* | 18.3 | -61.1 | 57.6 | 5.18 |
|  |  |  |  |  |  |  |  |  |  |  |  |
| Left Injury > Right Injury | |  |  |  |  | Left Injury > Right Injury | |  |  |  |  |
|  | *Posterior cingulate* | -3.8 | -6.1 | 35 | 67.78 |  | *Supramarginal* | 37.9 | -29.9 | 22 | 32.35 |
|  | *Supramarginal* | -38.9 | -37.5 | 18 | 14.32 |  | *Posterior cingulate* | 3.8 | -9.1 | 35.4 | 66.37 |
|  | *Lingual* | -8.5 | -67.5 | 3.2 | 1.22 |  | *Precentral* | 34.7 | -23.4 | 43.9 | 36.35 |
|  |  |  |  |  |  |  | *Parasopercularis* | 34.9 | 11.7 | 13 | 24.86 |
|  |  |  |  |  |  |  | *Rostral middle frontal* | 38.8 | 42.2 | -2.3 | 11.06 |
|  |  |  |  |  |  |  | *Precuneus* | 10.6 | -67 | 36.2 | 3.38 |

**Supplementary Table 2.** Cortical thickness evaluation of hemisphere. Brain regions showing group (Right ankle injury; Left ankle injury) differences in cortical thickness. Results are provided showing where brain regions are greater in the right ankle injury group than the left ankle injury and the opposite comparison. The size of each cluster is reported in mm^2^.
