# Supplementary material for: Biological and behavioral markers of pain following nerve injury in humans
Source: Neurobiol Pain. 2019 Dec 4;7:100038. doi: 10.1016/j.ynpai.2019.100038 (PMC6926375; doi:10.1016/j.ynpai.2019.100038)
Supplement: Supplementary data 3 [file mmc3.docx]

| **Pediatric Pain Screening Tool** | | | | | | | | | | | |
| --- | --- | --- | --- | --- | --- | --- | --- | --- | --- | --- | --- |
| Left Hemisphere | |  |  |  |  | Right Hemisphere | |  |  |  |  |
| Negative correlation | | Size (mm^2^) | x | y | z | Negative correlation | | Size (mm^2^) | x | y | z |
|  | Caudal middle Frontal | 142.46 | -36 | 23.1 | 46.7 |  |  |  |  |  |  |
|  | Lingual | 50.39 | -23.4 | -57 | 0.1 |  |  |  |  |  |  |
|  | Lateral orbitofrontal | 30.9 | -24.4 | 26.7 | -13.6 |  |  |  |  |  |  |
|  | Superior frontal | 17.84 | -16.5 | 62.2 | -0.6 |  |  |  |  |  |  |
|  | Supramarginal | 14.37 | -59.7 | -33.6 | 31.7 |  |  |  |  |  |  |
|  |  |  |  |  |  |  |  |  |  |  |  |
| Positive correlation | | Size (mm^2^) | x | y | z | Positive Correlation | | Size (mm^2^) | x | y | z |
|  | Pars opercularis | 68.23 | -49 | 12.7 | 2.9 |  | Lateral occipital | 163.98 | 31.3 | -85.4 | -13.2 |
|  | Entorhinal | 39.34 | -29.4 | -8.1 | -32.6 |  | Lateral orbitofrontal | 25.49 | 14.6 | 18.8 | -23.21 |
|  | Temporal pole | 11.75 | -28.8 | 3.6 | -32.3 |  |  |  |  |  |  |
|  | Pericalcarine | 39.26 | -16.4 | -95.9 | -2.4 |  |  |  |  |  |  |
|  | Paracentral | 9.49 | -5.3 | -17.1 | 51.2 |  |  |  |  |  |  |
|  | Superior parietal | 8.33 | -14.2 | -72.7 | 40 |  |  |  |  |  |  |
|  |  |  |  |  |  |  |  |  |  |  |  |
| **Fear of Pain Questionnaire** | | | | | | | | | | | |
| Left Hemisphere | |  |  |  |  | Right Hemisphere | |  |  |  |  |
| Negative Correlation | | Size (mm^2^) | x | y | z | Negative Correlation | | Size (mm^2^) | x | y | z |
|  | Lateral oribitofrontal | 111.67 | -26.1 | 25.7 | -15.3 |  | Lateral occipital | 87.53 | 33.6 | -83.3 | 0.3 |
|  | Fusiform | 93.19 | -35.8 | -32.1 | -16.8 |  | Inferior parietal | 87.44 | 44.2 | -72 | 13.9 |
|  | Superior frontal | 130.13 | -8.3 | 9.6 | 53.2 |  | Supramarginal | 38.85 | 46.3 | -40.3 | 38.2 |
|  | Insula | 113.03 | -31.6 | 17.1 | 1.5 |  | Superior parietal | 41.55 | 16.2 | -73.8 | 41.2 |
|  | Superior parietal | 83.48 | -23.7 | -60.1 | 61.6 |  | Inferior parietal | 46.72 | 53.1 | -56.6 | 11.3 |
|  | Precuneus | 54.4 | -10.4 | -57.2 | 30.3 |  | Superior parietal | 24.94 | 25.4 | -43.5 | 57.9 |
|  | Lateral occipital | 122.6 | -34.7 | -84.8 | -0.8 |  | Inferior parietal | 35.32 | 44.4 | -62.4 | -4.2 |
|  | Insula | 65.08 | -33.1 | -22 | 14.2 |  | Superior parietal | 32.17 | 43 | -76 | -0.5 |
|  | Lateral occipital | 289.27 | -27.6 | -83.2 | 12.2 |  | Inferior temporal | 39.74 | 20 | 47.8 | -12.5 |
|  | Superior parietal | 67.18 | -25.4 | -43.6 | 55.1 |  | Lateral occipital | 24.01 | 7.3 | -92.3 | -8.9 |
|  | Lingual | 76.98 | -24 | -56.4 | -5.2 |  | Lateral orbitofrontal | 15.93 | 39.5 | 6.9 | -28.1 |
|  | Rostral middle frontal | 31.14 | -40.2 | 26.4 | 15.2 |  | Superior temporal | 29.91 | 41.4 | -26.8 | 40.3 |
|  | Temporal pole | 23.3 | -38.5 | 6.7 | -36.7 |  | Pars Triangularis | 2.31 | 30 | 13.9 | -12.9 |
|  | Superior parietal | 31.34 | -22.7 | -63.6 | 30.3 |  | Supramarginal | 4.9 | 60.3 | -37.7 | 35.8 |
|  | Inferior parietal | 24.76 | -33.9 | -71 | 45.8 |  | Superior Temporal | 2.45 | 44.1 | -20.1 | -4.9 |
|  | Cuneus | 39.36 | -6.2 | -88.1 | 17.9 |  | Supramarginal | 0.75 | 48.2 | -29.1 | 38.4 |
|  | Caudal middle frontal | 4.72 | -29.9 | -0.9 | 47.6 |  |  |  |  |  |  |
|  | Isthmus cingulate | 4.49 | -8.5 | -46 | 30.2 |  |  |  |  |  |  |
|  |  |  |  |  |  |  |  |  |  |  |  |
| Positive Correlation | | Size (mm^2^) | x | y | z | Positive Correlation | | Size (mm^2^) | x | y | z |
|  | Temporal pole | 50.86 | -26 | 6.3 | -38.6 |  | Superior frontal | 45.32 | 14.6 | 16.1 | 58.8 |
|  | Inferior temporal | 19.77 | -55.8 | -52.2 | -11.5 |  | Lingual | 46 | 50.2 | 32.8 | -6 |
|  | Precentral | 4 | -45.7 | -2 | 33.3 |  |  |  |  |  |  |
|  |  |  |  |  |  |  |  |  |  |  |  |
| **Pain Catastrophizing Scale** | | | | | | | | | | | |
| Left Hemisphere | |  |  |  |  | Right Hemisphere | |  |  |  |  |
| Negative correlation | | Size (mm^2^) | x | y | z | Negative correlation | | Size (mm^2^) | x | y | z |
|  | Superior parietal | 96.46 | -22.2 | -39.3 | 55.6 |  | Superior parietal | 47.89 | 17.1 | -73.6 | 39.3 |
|  | Insula | 52.22 | -33.2 | 17.8 | -3.1 |  | Superior temporal | 36.3 | 40.9 | 5.8 | -26.3 |
|  | Superior parietal | 121.04 | -25.6 | -77.4 | 15.2 |  | Superior frontal | 23.44 | 9.7 | 8.5 | 48.4 |
|  | Superior parietal | 31.22 | -23.9 | -63.3 | 29.5 |  | Superior temporal | 2.55 | 45 | -18.4 | -6.2 |
|  | Lateral occipital | 61.09 | -34.4 | -85 | -1.4 |  |  |  |  |  |  |
|  | Caudal middle frontal | 44.1 | -34.5 | 23.4 | 46.8 |  |  |  |  |  |  |
|  | Insula | 34.07 | -33.1 | -21.9 | 15.4 |  |  |  |  |  |  |
|  | Inferior parietal | 20.4 | -3.4 | -70.4 | 45.3 |  |  |  |  |  |  |
|  | Lateral orbitofrontal | 25.45 | -26.7 | 27 | -15.1 |  |  |  |  |  |  |
|  | Superior frontal | 18.91 | -8.2 | 8.9 | 53.1 |  |  |  |  |  |  |
|  | Superior parietal | 0.5 | -29.7 | -62.8 | 40.2 |  |  |  |  |  |  |
|  | Temporal Pole | 1.84 | -38.9 | 6.8 | -36.4 |  |  |  |  |  |  |
|  |  |  |  |  |  |  |  |  |  |  |  |
| Positive correlation | | Size (mm^2^) | x | y | z | Positive correlation | | Size (mm^2^) | x | y | z |
|  | Temporal Pole | 10.07 | -31 | 3.9 | -31.1 |  | Rostral middle frontal | 43.35 | 34.9 | 16.6 | 25 |
|  | Lateral Orbitofrontal | 12.2 | -19.1 | 47.7 | -13.6 |  | Superior Frontal | 75.43 | 15.2 | 16.3 | 58.6 |
|  |  |  |  |  |  |  | Lateral Orbitofrontal | 53.6 | 15.6 | 17.6 | -20.3 |
|  |  |  |  |  |  |  | Superior Frontal | 0.39 | 23.3 | 14.4 | 45.2 |

**Supplementary Table 3**. Cortical thickness and self-report questionnaires. Brain regions showing a significant (p<0.05) correlation with self-report questionnaires (Pediatric Pain Screening Tool; Fear of Pain Questionnaire; Pain Catastrophizing Scale). The cluster size is reported in mm^3^ and the coordinates of the clusters max is provided in Talairach coordinates.
